# Seroprevalence of Hepatitis A, B and C Among a Sample of Refugees in Egypt: An Exploratory Survey

**DOI:** 10.1007/s44197-022-00060-6

**Published:** 2022-09-15

**Authors:** Engy Mohamed El-Ghitany, Ayat Ashour, Marwa M. Fekry, Ehab Elrewany, Azza Galal Farghaly, Eman A. Omran

**Affiliations:** 1 Department of Tropical Health, High Institute of Public Health, Alexandria University, 165 El-Horreya Avenue–El-Ibrahimia, Alexandria, Egypt; 2 Department of Family Health, High Institute of Public Health, Alexandria University, Alexandria, Egypt; 3 Department of Microbiology, High Institute of Public Health, Alexandria University, Alexandria, Egypt

**Keywords:** HCV, HBV, HAV, Refugees, Immigrants, Seroprevalence

## Abstract

**Background:**

Estimating the prevalence of infectious diseases, including viral hepatitis, among refugees is important for evaluating their health needs and predicting the burden on the health system of the host country. This study aimed at estimating the seroprevalence of viral hepatitis among refugees in Egypt.

**Methods:**

This cross-sectional study involved a heterogeneous group of 501 refugees. Enzyme-linked immunosorbent assays were used to detect IgG antibodies against hepatitis A virus (HAV), B virus (HBV) surface antigen (anti-HBsAg), C virus (HCV), and HBV surface antigen (HBsAg).

**Results:**

Anti-HAV was the most prevalent marker (*n* = 482, 96.2%), followed by anti-HBs (*n* = 142, 28.3%) and HBsAg (*n* = 21, 4.2%), while only four refugees (0.8%) had positive anti-HCV IgG. Anti-HBs was higher in males (*p* < 0.05). Older refugees and non-working subjects had significantly higher seropositive rates of anti-HAV (*p* = 0.051 and *p* = 0.023, respectively), while students and those below 15 years of age had higher rates of anti-HBs (*p* < 0.05). Positive HBsAg results were associated with history of hepatitis (*p* < 0.001). Obese participants were more likely to be positive for HBsAg (*p* = 0.025) and anti-HBs (*p* < 0.05). Sudanese refugees had significantly higher rates of anti-HAV antibodies (p = 0.049), while Yemini refugees had significantly higher rates for HBsAg (*p* = 0.019) positivity. Residents of Dakahlia had significantly higher rates of anti-HAV (*p* = 0.008) and anti-HBs (*p* < 0.05). None of the studied risk factors was significantly associated with anti-HCV.

**Conclusion:**

Refugees in Egypt have poor immunity against HBV with intermediate to high HBV and low HCV prevalence rates. Despite that 65% of refugees received the HAV vaccine, almost all had IgG anti-HAV, denoting previous infection.

## Introduction

As of 31 January 2022, the refugee population registered in Egypt by the United Nations High Commissioner for Refugees (UNHCR) comprised 137,599 Syrians, representing almost 50% of all refugees in Egypt, while the remaining refugees were from Sudan, Eritrea, Ethiopia, South Sudan and more than 50 other nations [1]. In Egypt, refugees mostly reside in urban areas across the country and are mainly concentrated in Giza, Cairo, followed by Alexandria. Refugees continue to have access to public education and health services at the same scale as Egyptians [2].

Neighbouring countries that provide asylum and safe shelter for those who migrate are witnessing increased demographic and economic hardships. Population mobility is also associated with the introduction of new diseases in the host community, including infections of the respiratory tract, diarrhoeal diseases, tuberculosis, hepatitis A, B and C (HAV, HBV and HCV, respectively) and others [3]. Fleeing of refugees from their countries might interrupt the completion of their vaccination schedule against some of the vaccine-preventable diseases such as HBV. Moreover, the living conditions of such individuals might often involve overcrowding and inadequate hygienic practices, which might in turn raise their odds of contracting infectious diseases in their host country [4, 5].

Between 1990 and 2013, the worldwide reported mortalities from viral hepatitis had surged from 0.89 million to 1.45 million [6]. With an estimated 8–10 million persons living with viral hepatitis in Egypt alone, viral hepatitis represents a significant public health concern in the country [7]. In 2015, Egypt’s HCV infection prevalence of 7% in adults was among the highest in the world and accounted for 7.6% of the country’s mortality [8]. Between 2014 and 2020, Egypt screened more than 50 million and treated more than 4 million residents for HCV [8].

Identification of the prevalence of infectious diseases among refugees is important for estimating their health needs, predicting the burden on the health system and accurately planning the response at all different public health levels [4]. Since viral hepatitis is a major health problem in Egypt, the estimation of its magnitude among refugees would help in estimating any additional disease burden as well as provision of adequate prevention and control measures for such individuals.

This study aimed at estimating the seroprevalence of hepatitis A, B and C viruses among a sample of refugees in Egypt.

## Methods

The study was conducted in different Syrian and migrants/refugees community centres in Giza, Alexandria, Dakahlia and Damietta governorates. These centres provide services and activities for Syrians such as women empowerment, workshops on how to run a business and improve marketing skills, educational sessions for children and awareness sessions for public on topics such as health and law.

Refugees of both genders with no age restrictions were recruited by inviting all those attending any of the study settings during the data collection days. Migrants should have been residing in Egypt for at least 6 months.

The sample size was calculated to be 385 refugees at a 95% confidence level with a 5% margin of error. The number of recruited participants was 501 from different age groups of both genders.

### Data Collection Methods and Tools

After obtaining written consent from each participant, a pre-designed structured questionnaire was filled out by the interviewer to collect the following: socio-demographic data, date since fleeing their home country and entering Egypt, history of viral hepatitis and vaccination history for HBV and HAV. The questionnaire was piloted to test for its validation [9, 10].

This included four parameters:

- *Face validation*: to ensure that the respondents’ understanding of the questions was aligned with our goals.
- *Pilot data preparation*: in this step, we ensured that response bias was minimized and the possibility of predicting missing data was investigated.
- *Content validation*: in this step, we verified that the question items targeted the aim of the study.
- *Content reliability*: in this step, we investigated the relevance of the question items.

Weight was measured by a calibrated weighing scale. The height of each participant was measured and recorded to calculate the body mass index (BMI) of each participant. Weight (kg) and height (cm) were obtained using a standard office scale. BMI = kg/m^2^. Obesity was defined according to age; adults were considered obese if BMI > 30 kg/m^2^ and for children (< 18 years) a BMI equal to or greater than the 95th percentile was considered obese [11].

### Laboratory Tests

Five millilitres of blood was drawn from all participants and transferred to an ice box without delay to be processed at the laboratory of the High Institute of Public Health, Alexandria University. To separate the serum, 5000 rpm centrifugation was done. Sera aliquots were stored at − 20 °C until used for detection of anti-HAV, HBsAg, anti-HBs and anti-HBc by enzyme-linked immunosorbent assay (ELISA) (kits manufactured by Precheck Bio, INC., CA, USA). Correlation of HBV protection was considered when anti-HBs IgG exceeded 10 mIU/mL [12].

### Statistical Analysis

Data were extracted, revised, coded and fed to statistical software IBM SPSS version 25 (SPSS, Inc. Chicago, IL). All statistical analysis was done using two-tailed tests. *P* values less than 0.05 were statistically significant. Qualitative data were described using frequency and percentage, while quantitative data were described using range (minimum and maximum), mean, and standard deviation. The Chi-square test was used to test the association between categorical variables, while Fisher’s exact or Monte Carlo correction was used as a correction for Chi-square when more than 20% of the cells have an expected count of less than 5.

## Results

A total of 501 participants were included, the majority of whom were 35–44 years old (*n* = 115, 23.0%). Females constituted 68.1% (*n* = 341), and 41.7% (*n* = 209) of all participants were housewives. The majority of adult participants received secondary education (*n* = 116, 29.7%) or university degree (*n*= 112, 28.6%). Syrians were the most represented nationality (*n* = 332, 66.3%). Alexandria was the main represented governorate (*n* = 203, 40.5%), and most participants (*n* = 213, 42.5%) reported duration of stay of 7–9 years. Most participants (*n* = 333, 66.5%) reported receiving HBV vaccines and 326 (65.1%) received HAV vaccines. Obesity was prevalent in 30.3% of the participants. Only six participants (1.2%) reported a history of viral hepatitis. Some participants did not know whether they were vaccinated or not against HAV (29.7 %) and HBV (28.7 %) (Table 1).

**Table 1:** General characteristics of 501 refugees in Egypt

	Total (*n* = 501)	
	No	%
Age (years)		
< 15	66	13.2
15–24	90	18.0
25–34	76	15.2
35–44	115	23.0
45–54	90	18.0
55 +	64	12.8
Min–max	5–85	
Mean ± SD	35.3 ± 16.56	
Gender		
Male	160	31.9
Female	341	68.1
Marital status		
Single/child	175	34.9
Married	283	56.5
Divorced	22	4.4
Widowed	21	4.2
Education (for adults older than 18 yrs, *n* = 391)		
Illiterate	14	3.6
Primary	59	15.1
Preparatory	90	23
Secondary	116	29.7
University	112	28.6
Occupation		
Student	131	26.1
Housewife	209	41.7
Not working	51	10.2
Manual labourer	67	13.4
Professional job	43	8.6
Nationality		
Syrian	332	66.3
Sudanese	64	12.8
Yemeni	26	5.2
Eritrean	66	13.2
Others	13	2.6
Current governorate of residence in Egypt		
Alexandria	203	40.5
Giza	164	32.7
Dakahlia	67	13.4
Damietta	67	13.4
Duration of living in Egypt (years)		
< 1	26	5.2
1–3	117	23.4
4–6	101	20.2
7–9	213	42.5
> 9	44	8.8
History of receiving HBV vaccine		
No	24	4.8
Yes	333	66.5
Do not know	144	28.7
History of receiving HAV vaccine		
No	26	5.2
Yes	326	65.1
Do not know	149	29.7
Obesity		
No	349	69.7
Yes	152	30.3
History of viral hepatitis		
No	495	98.8
Yes	6	1.2

The majority of participants (69.5%) were positive for only one of the three tested antibodies, 27.3% were positive for two antibodies, and 0.4% were positive for all three antibodies, while 2.8% were negative for all of the three viral antibodies.

As seen in Table 2, the most commonly positive viral hepatitis marker was anti-HAV IgG (*n* = 482, 96.2%), followed by anti-HBs IgG (*n* = 142, 28.3%) and then HBsAg (*n* = 21, 4.2%), while only four participants (0.8%) were positive for anti-HCV IgG.

**Table 2:** Prevalence of viral hepatitis among a sample of refugees in Egypt

	Total (*n* = 501)	
	*N*	%
Anti-HAV IgG		
Negative	19	3.8
Positive	482	96.2
HBsAg		
Negative	480	95.8
Positive	21	4.2
Anti-HBs IgG		
Negative	359	71.7
Positive	142	28.3
Anti-HBs IgG		
Negative	359	71.7
Positive	142	28.3
Anti-HCV IgG		
Negative	497	99.2
Positive	4	0.8

Concerning the titre of anti-HBs, the majority of participants (*n* = 359, 71.7%) had titres < 10 mIU/mL, while 74 participants (14.8%) had titres ranging between 10 and 100 mIU/mL, and only 13.6% of participants (*n* = 68) had titres exceeding 100 mIU/mL.

Among the 501 participants, the majority (*n* = 343) were negative for both anti-HBs and HBsAg, representing 71.5% of all HBsAg-negative tests (*n* = 480), while 137 participants were positive for anti-HBs but negative for HBsAg (28.5%). Among the 21 participants with positive HBsAg, 16 (76.2%) were negative for anti-HBs, while 5 (23.8%) were also positive for anti-HBs. The five cases positive for both HBsAg and anti-HBs had a mean age of 25 years, two were males and three females, three Syrians and two Sudanese and all were anti-HCV negative and four of them were anti-HAV positive. Four of them received the HBV vaccine and one participant was not sure whether he received it or not. None of them were smokers. The titre of their anti-HBs was 10–100 mIU/mL in two of them, while the other three participants had titres exceeding 100 mIU/mL

Regarding HBsAg seropositivity, significantly higher rates were reported among Yemini participants (11.5%) followed by Sudanese (10.9%), while the lowest rates were among Eritreans (1.5%), *p* = 0.019. There was a significant difference in HBsAg positivity in relation to the duration of stay in Egypt (*p* = 0.024). Receiving the HBV vaccine was not associated with HBsAg positivity. Obese participants and those who reported a history of viral hepatitis were more likely to be positive for HBsAg (*p* = 0.025, *p* = 0.000, respectively) (Table 3). Of the 21 HBsAg-positive participants, only three had a history of viral hepatitis (14.3%)

**Table 3:** Distribution of HBsAg among a sample of refugees in Egypt

	HBsAg				Test of significance
	Positive		Negative		(*p* value)
	No	%	No	%	
Age (years)					
Less than 15	2	3	64	97	
15–24	3	3.3	87	96.7	Fisher exact = 3.742
25–34	2	2.6	74	97.4	*p* = 0.514
35–44	7	6.1	108	93.9	
45–54	6	6.7	84	93.3	
55 +	1	1.6	63	98.4	
Gender					
Male	6	3.8	154	96.3	*X*^2^ = 0.114
Female	15	4.4	326	95.6	*p* = 0.735
Nationality					
Syrian	9	2.7	323	97.3	
Sudanese	7	10.9	57	89.1	Fisher exact = 12.835
Yemeni	3	11.5	23	88.5	*p* = 0.019
Eritrean	1	1.5	65	98.5	
Others	1	7.7	12	92.3	
Occupation					
Student	6	4.6	125	95.4	
Housewife	9	4.3	200	95.7	Fisher exact = 0.461
Not working	2	3.9	49	96.1	*p* = 0.99
Manual labourer	2	3	65	97	
Professional job	2	4.7	41	95.3	
Current governorate of residence in Egypt					
Alexandria	11	5.4	192	94.6	Fisher exact = 1.778
Dakahlia	1	1.5	66	98.5	*p* = 0.528
Damietta	2	3	65	97	
Giza	7	4.3	157	95.7	
Duration of living in Egypt (years)					
< 1	3	11.5	23	88.5	
1–3	2	1.7	115	98.3	Fisher exact = 11.069
4–6	8	7.9	93	92.1	*p* = 0.024
7–9	5	2.3	208	97.7	
>9	3	6.8	41	93.2	
History of having HBV vaccine					
No	3	12.5	21	87.5	*X*^2^ = 4.412
Yes	12	3.6	321	96.4	*p* = 0.11
Do not know	6	4.2	138	95.8	
History of viral hepatitis					
No	18	3.6	477	96.4	*X*^2^ = 31.731
Yes	3	50	3	50	*p* = 0.000
Obesity					
No	10	2.9	339	97.1	*X*^2^ = 5.039
Yes	11	7.2	141	92.8	*p* = 0.025

Higher seropositivity rates of anti-HBs IgG were noted among refugees younger than 15 years of age (56.1%, *p* < 0.001), students (51.9%, *p* < 0.001), males (36.3%, *p* < 0.01), obese individuals (81.6%, *p* = 0.001) and refugees residing in Dakahlia (41.8%, *p* = 0.025) (Table 4). Of the 142 participants with positive anti-HBs IgG, only one had a history of viral hepatitis. Receiving the HBV vaccine was not associated with anti-HBs IgG positivity or titre (data not shown)

**Table 4:** Distribution of anti-HBs IgG among a sample of refugees in Egypt

	Anti-HBs IgG				*p* value
	Positive		Negative		
	No	%	No	%	
Age (years)					
Less than 15	37	56.1	29	43.9	
15–24	43	47.8	47	52.2	< 0.001
25–34	20	26.3	56	73.7	
35–44	13	11.3	102	88.7	
45–54	14	15.6	76	84.4	
55 +	15	23.4	49	76.6	
Gender					
Male	58	36.3	102	63.8	< 0.01
Female	84	24.6	257	75.4	
Nationality					
Syrian	95	28.6	237	71.4	
Sudanese	24	37.5	40	62.5	
Yemeni	7	26.9	19	73.1	0.248
Eritrean	14	21.2	52	78.8	
Others	2	15.4	11	84.6	
Occupation					
Student	68	51.9	63	48.1	
Housewife	37	17.7	172	82.3	< 0.001
Not working	16	31.4	35	68.6	
Manual labourer	16	23.9	51	76.1	
Professional job	5	11.6	38	88.4	
Current governorate of residence in Egypt					
Alexandria	46	22.7	157	77.3	
Dakahlia	28	41.8	39	58.2	0.025
Damietta	20	29.9	47	70.1	
Giza	48	29.3	116	70.7	
Duration of living in Egypt (years)					
< 1	8	30.8	18	69.2	
1–3	38	32.5	79	67.5	
4–6	23	22.8	78	77.2	0.486
7–9	58	27.2	155	72.8	
> 9	15	34.1	29	65.9	
History of having HBV vaccine					
No	5	20.8	19	79.2	
Yes	103	30.9	230	69.1	0.187
Do not know	34	23.6	110	76.4	
Obesity					
No	235	67.3	114	32.7	0.001
Yes	124	81.6	28	18.4	
History of viral hepatitis					0.523
No	141	28.5	354	71.5	
Yes	1	16.7	5	83.3	

Only four participants (0.8%) were positive for anti-HCV and none of them gave a history of viral hepatitis. Those four anti-HCV seropositive participants had negative HBsAg results. Twenty-one had HBsAg-positive results and 4 had anti-HCV IgG

Higher seropositive rates of anti-HAV IgG were recorded among participants who were older in age (*p* = 0.051), Sudanese (100%, *p* = 0.049), and not working (compared to students) *p* = 0.023 and residents of Dakahlia compared to Giza (100% versus 92.1%, *p* = 0.008). Receiving the HAV vaccine was not associated with anti-HAV IgG positivity (Table 5). Of the 482 participants with positive anti-HAV, only 6 reported a clinical history of hepatitis (1.24%)

**Table 5:** Distribution of anti-HAV IgG among a sample of refugees in Egypt

	Anti-HAV IgG				*p* value
	Positive		Negative		
	No	%	No	%	
Age (years)					
Less than 15	60	90.9	6	9.1	
15–24	84	93.3	6	6.7	
25–34	73	96.1	3	3.9	0.051
35–44	113	98.3	2	1.7	
45–54	89	98.9	1	1.1	
55 +	63	98.4	1	1.6	
Gender					
Male	152	95	8	5	0.332
Female	330	96.8	11	3.2	
Nationality					
Syrian	321	96.7	11	3.3	
Sudanese	64	100	0	0	
Yemeni	23	88.5	3	11.5	0.049
Eritrean	61	92.4	5	7.6	
Others	13	100	0	0	
Occupation					
Student	120	91.6	11	8.4	
Housewife	205	98.1	4	1.9	
Not working	51	100	0	0	0.023
Manual labourer	65	97	2	3	
Professional job	41	95.3	2	4.7	
Current governorate of residence in Egypt					
Alexandria	198	97.5	5	2.5	
Dakahlia	67	100	0	0	
Damietta	66	98.5	1	1.5	0.008
Giza	151	92.1	13	7.9	
Duration of living in Egypt(years)					
< 1	26	100	0	0	
1–3	110	94	7	6	
4–6	95	94.1	6	5.9	0.242
7–9	208	97.7	5	2.3	
> 9	43	97.7	1	2.3	
History of having HAV vaccine					
No	26	100	0	0	
Yes	312	95.7	14	4.3	0.515
Do not know	144	96.6	5	3.4	
Obesity					
No	333	95.4	16	4.6	0.16
Yes	149	98	3	2	
History of viral hepatitis					
No	476	96.2	19	3.8	0.625
Yes	6	100	0	0	

There was no statistical association in the seropositivity between the anti-HAV IgG or anti-HBV IgG. The low number of anti-HCV IgG positive cases did not allow statistical analysis of their associated risk factors.

## Discussion

There is a disparity among countries and overall scarce information regarding the presence of national guidelines for screening infectious diseases among migrants and refugees [13]. Of the countries in the European Union and European Economic Area, 16 (59%) had implemented screening programmes and 15 (56%) had national guidelines [6]. No such guidelines are available in Egypt, and to the best of the authors’ knowledge, no such studies on refugees in Egypt have been published.

In our study, Syrians were the most represented nationality (*n* = 332, 66.3%), and this is in agreement with the large contribution of Syrians among all refugees in Egypt. Alexandria was the main represented governorate in our study (*n* = 203, 40.5%), and most participants (*n* = 213, 42.5%) reported a duration of stay of 7–9 years, which is in line with the time point at which the war in Syria erupted, 12 years ago.

Only 2.8% of our study participants were negative for antibodies against the three viruses: HAV, HBV and HCV, indicating that the majority of participants (97.2%) were exposed to at least one of the three tested viruses, either through infection or vaccination. This underscores the importance of studying these highly prevalent hepatic serum markers for appropriate prevention and control. In contrast to the high seroprevalence of viral hepatitis markers, a very low percentage of our participants (1.2%, *n* = 6) reported a history of viral hepatitis (diagnosed clinically, radiologically and by laboratory tests). This might be attributed to the seropositivity due to vaccination, or that clinical infections were asymptomatic or mild in nature, or due to recall bias of some participants who might have under-reported their viral hepatitis infections or vaccination status. More than a quarter of our participants did not know whether they were vaccinated or not against HAV (29.7%) and HBV (28.7%).

In our study, anti-HAV was the most prevalent serum marker for hepatitis (*n* = 482, 96.2%), denoting either previous vaccination (65.1% of participants had a history of receiving HAV vaccine) or previous HAV infection, whether clinical or sub-clinical. Despite the high seroprevalence of anti-HAV, only six participants reported a history of viral hepatitis (1.24%), indicating a mild course of infection.

The anti-HAV seroprevalence rate reported in this study among refugees is higher than that reported among Egyptians in a recent meta-analysis (71.5%) [14]. This might be due to the higher prevalence of HAV in countries of origin of those refugees, or due to the deteriorated sanitary and housing conditions of refugees, or since the majority of participants in our study were adults rather than children. The reported anti-HAV seroprevalence rates in a meta-analysis from Syria and Yemen were 88.8 and 86.6%, respectively [14], and 100% in a study on Eritrean asylum seekers residing in the Netherlands [15]. The high seroprevalence of anti-HAV IgG indicates that those refugees are immune to future HAV infections and that they pose little or no risk of HAV transmission to the Egyptian community.

In developing countries, immunity against HAV is acquired through infection rather than vaccination, as this infection is endemic in most African countries [16]. The vaccine is not among the Expanded Programme on Immunization (EPI) of Egypt and most developing countries [16] (probably due to its cost and/or unavailability and good prognosis of the natural infection). Immigrants and travellers from areas of low disease rates are therefore at high risk of infection when residing in countries where the infection is endemic [17]. Hepatitis A infection is transmitted through the faeco-oral route and in conditions with reduced sanitary conditions, such as during natural disasters and wars. On September 9, 2018, the Governorate of Aleppo, Syria, informed the World Health Organization office in Syria that internally displaced persons (displaced since early 2018) in the northwestern and western parts of Aleppo were experiencing a suspected HAV outbreak, with a total of 638 cases of HAV infection. A high displacement rate, deteriorated sanitary and health conditions and poor water quality likely contributed to this outbreak [17].

In our study, higher anti-HAV seropositivity was recorded among participants who were older in age and this was statistically close to significance (*p* = 0.051). Kaddoura et al. reported that among internally displaced Syrians who suffered an outbreak of HAV disease, 98.6% of them were below 15 years of age [17]. In Egypt, 50% or more of the population develop immunity by the age of 15 years [16]. Collectively, this data indicates contracting HAV infections during childhood, with higher seropositivity developing with older age. However, there has been a recent change in the age distribution of HAV infections, shifting to older age groups, making them more prone to severe clinical presentations and complications [14, 16, 18].

In the present study, other factors associated with seropositivity of anti-HAV IgG were being Sudanese (100%, *p* = 0.049), not working (compared to students, *p* = 0.023) and among residents of Dakahlia (*p* = 0.008). Higher seroprevalence might indicate more frequent environmental exposure to HAV among those risk groups.

Despite that 66.5% of participants reported receiving HBV vaccines, anti-HBs was positive in only 28.3% of them. The low seroprevalence rate despite higher reported vaccination rates may reflect waning immunity due to the lapse of a long time since vaccination, vaccination failure or the possibility of recall bias. Incomplete vaccine schedules might have led to lower immune response. Administration of three doses of HBV vaccine during childhood is known to be almost 95% effective in preventing infection [19, 20]. The HBV vaccine has been part of the compulsory childhood vaccination programme in Egypt since 1992 and has resulted in a dramatic drop in HBV infections ever since, evidenced by decreased seroprevalence of HBsAg (in which HBsAg-positive individuals are potentially infectious carriers), and a decreased seroprevalence of HBV anti-core antibodies (anti-HBcAb) (which indicates HBV infection) [19]. Unfortunately, in our study anti-HBcAb was not done, so it was not possible to determine whether cases with positive anti-HBs were vaccinated or previously infected. Nevertheless, the importance of these cases lies in them being potentially infectious to others and thus requiring treatment and adoption of prevention and control measures (including vaccination) for their contacts.

According to the UNICEF estimates of immunization coverage in 2020, the percentage of surviving infants who received the three doses of HBV vaccine following the birth dose was 95% in Eritrea, 94% in Egypt, 90% in Sudan, 72% in Yemen and only 49% in Syria [21]. Since most of our study participants were from Syria (66.3%), it is noteworthy to mention that since the introduction of HBV in their EPI vaccination programme, low rates of HBsAg (5.6%) were reported in the following years. Unfortunately, since the war in Syria began in 2011, vaccination rates have fallen significantly with the subsequent rise of HBV infections among the younger Syrian generations who have not received their childhood vaccinations [18]. The discontinuation of vaccine schedules in countries with conflicts raises hazards of vaccine-preventable diseases among refugees in host countries.

In our study, concerning the titre of anti-HBs, only 74 participants (14.8%) had a titre ranging between 10 and 100 mIU/mL, and only 13.6% of participants (*n* = 68) had a titre exceeding 100 mIU/mL. The declining rate of anti-HBs seroprotection with age is reported to reach its lowest level in individuals aged 11–20 years [22]. Anti-HBs IgG concentrations between 10 and 100 IU/L indicate a low level of immune protection against HBV, whereas concentrations > 100 IU/L indicate a high level of protection [23]. Consequently, we could conclude that there was a high risk of infection among the majority of participants in our study. This necessitates vaccination of these refugees against HBV to protect them, their families and the Egyptian community.

In the present study, the seroprevalence of HBsAg was 4.2% (*n* = 21), which was higher than the rate reported by the Egyptian Health Issues Survey, 2015, in a large population-based study (1%) [8]. Refugees from Syria had the lowest seroprevalence of HBsAg (2.7%) compared to Sudanese (10.9%) and Yemeni (11.5%) refugees. Comparable seroprevalence rates have been published in a meta-analysis in Sudanese (9.76%), Syrian (2.62%), Yemeni (8·38%) and Eritrean (2.49%) populations [25], denoting that the seroprevalence rates in the present study reflect the rates in the country of origin of refugees, rather than their host country. In our study, the rates of HBsAg among refugees of all nationalities were higher than the rates reported in the Egyptian community, which poses a risk of transmission of HBV to the community, thus mandating treatment and vaccination of refugees to reduce such risk.

There was no significant difference between Egyptian governorates regarding HBsAg (*p* = 0.528) and this was consistent with another study [Elsabawy, 2011 #31]. However, there was a significant difference in anti-HBsAg among governorates (*p* = 0.025), where Dakahlia Governorate had the highest anti-HBsAg (41.8%) but the lowest HBsAg, denoting probable higher vaccination of this group of refugees.

Among our 501 participants, the majority (*n* = 343) were negative for both anti-HBs IgG and HBsAg, representing 71.5% of all HBsAg-negative tests (*n* = 480), while 137 participants were positive for anti- HBs IgG but negative for HBsAg (28.5%). Among the 21 participants with positive HBsAg, 16 (76.2%) were negative for anti-HBs IgG, while 5 (23.8%) were also positive for anti-HBs. These five cases might be cases of chronic active hepatitis. A review study reported that the prevalence of concurrent HBsAg/anti-HBs in patients with HBV infection can vary from less than 5% to almost 30%, and was usually associated with chronic active hepatitis. Mutations in the viral genomes, immune status and genetic factors of hosts may contribute to the concurrent coexistence of HBsAg/anti-HBs [24].

Obese participants were more likely to be positive for HBsAg (*p* = 0.025), denoting a higher risk of complications among obese individuals. This was also reported in other studies and was attributed to impaired dendritic cell functions among obese subjects, leading to decreased anti-HB surface and core antigen-specific immune responses [26].

Higher positivity rates of anti-HBs were noted among: refugees younger than 15 years of age (56.1%, *p* =  < 0.001), students (51.9%, *p* < 0.001), males (36.3%, *p* < 0.01), obese individuals (81.6%, *p* = 0.001) and refugees residing in Dakahlia (41.8%, *p* = 0.025). Young participants (< 15 years), who were most probably students, had higher positivity rates, which might have been due to the relatively shorter time lapse between receiving HBV vaccine and testing. Regarding residence, however, there was no significant difference between governorates regarding HBsAg (Fisher exact = 1.778, *p* = 0.528). This was consistent with another study which reported that the morbidity rate of HBV was not significantly different between populations in Egyptian governorates. However, there was a significant difference in anti-HBsAg among governorates (*p* = 0.025), where Dakahlia Governorate had the highest anti-HBsAg (41.8%) but the lowest HBsAg, denoting probable higher vaccination of this group of refugees.

In our study, only four participants (0.8%) were positive for anti-HCV, but none of them gave a history of viral hepatitis. These cases should be considered as presumptively infected with HCV. A repeatedly reactive result is consistent with current or past HCV infection; however, a positive PCR test to detect viral RNA test should have been done to confirm a current infection [Control, 2017 #30]. These cases might thus pose a public health hazard, owing to their probable infectivity. Our seroprevalence of anti-HCV is extremely lower than the rates in the Egyptian community reported by the Egyptian Demographic and Health Surveys (2015), where the total seroprevalence in those aged < 60 years was 6.3% [7]. A study in 2017 involving 21 Egyptian governorates reported an overall anti-HCV seroprevalence of 14.8% [27]. The low seroprevalence of anti-HCV in refugees in our study might reflect the lower HCV rates in their countries compared to rates in Egypt.

In a meta-analysis on refugees from intermediate and high endemic countries, the overall pooled anti-HCV seroprevalence in migrants was 1.9% (1.4–2.7%) [13], which is comparable to the rate reported in our group of refugees. Differences in prevalence rates of viral hepatitis among studies might be attributed to the composition of the migrant/refugee study population where the studies were carried out [15]. In March 2019, refugees, asylum seekers and migrants in Egypt were included in the national campaign “100 Million Seha (health)” campaign, which offered free screening and treatment against HCV, and by the end of September 2019, 30,000 refugees and migrants underwent the screening of HCV all over Egypt [28]. In our study, positive anti-HCV results were not associated with any of the study's factors, probably due to the small number of positive anti-HCV individuals. A study by El-Ghitany et al. reported that a higher prevalence of anti-HCV was found among males, older age and in rural residence [27].

In the present study, a higher prevalence of HAV compared to HBV and HCV among refugees is consistent with a large Egyptian study, which compared the prevalence of the three viral infections to rates reported by surveillance from 2001 to 2004, and found a significant increase in the proportion of HAV infections from 40.2 to 89.7% with reductions in the proportions of HBV and HCV infections from 30.0 to 2.8% and 29.8 to 3.0%, respectively [29]. Therefore, the pattern of viral infections among refugees in our study is similar to that in the Egyptian community. Public health efforts towards refugees regarding viral hepatitis should thus be targeted in the same manner as in the Egyptian community.

Some imported infectious diseases (including viral hepatitis) may remain asymptomatic for months or years, whilst early diagnosis and treatment may prevent complications in later life and break the cycle of transmission in the host country [5]. Consequently, the burden on the health system secondary to viral hepatitis should extend to possible complications of cirrhosis and hepatocellular carcinoma in untreated cases. This underscores the importance of early screening of such infections among refugees, especially when we consider their financial and social constraints in host countries, which should not be further complicated by health-related problems.

## Conclusion

Despite that 65% of refugees received the HAV vaccine, almost all had IgG anti-HAV, denoting previous infection. HBsAg had a lower seropositivity rate (*n* = 21, 4.2%) and only four refugees (0.8%) had positive anti-HCV IgG. Different socio-demographic and clinical factors were associated with each of anti-HAV, anti-HBs IgG and HBsAg. Screening of HBV among refugees is recommended along with vaccination of seronegative individuals.

### Limitations

Our study included a majority of Syrian refugees, and refugees from other nationalities might have been under-represented. Moreover, testing for HBV in our study should have been coupled with testing for anti-HBc IgG to differentiate whether positive results of HBsAg were due to acute or chronic HBV infections. Moreover, cases with positive anti-HCV IgG should have been confirmed as current HCV infection by performing PCR test. Another limitation of our study was the recall bias faced during data collection, particularly the history of vaccination and viral hepatitis.

## Data Availability

Available and kept confidentially.

## Abbreviations

Ag: Antigen
BMI: Body mass index
EEA: European economic area
ELISA: Enzyme-linked immunosorbent assay
EPI: Expanded Programme on Immunization
EU: European Union
HAV: Hepatitis A virus
HBV: Hepatitis B virus
HCV: Hepatitis C virus
IgG: Immunoglobulin G
UNHCR: United Nations High Commissioner for Refugees
UNICEF: United Nations Children’s Fund

## References

[1] United Nations High Commissioner for Refugees (UNHCR) E. UNHCR Egypt Monthly Statistical Report 2022 [cited 2022. Available from: chrome-extension://efaidnbmnnnibpcajpcglclefindmkaj/viewer.html?pdfurl=https%3A%2F%2F. www.unhcr.org

[2] United Nations High Commissioner for Refugees (UNHCR) E. Egypt: 3RP Regional Refugee & Resilience Plan in Response to the Syria Crisis (2020/2021) 2021 [Available from: https://www.unhcr.org/eg/wp-content/uploads/sites/36/2020/10/3RP2021EN.pdf

[3] Shetty AK (2019). Infectious diseases among refugee children. Children.

[4] Gushulak BD, MacPherson DW (2004). Globalization of infectious diseases: the impact of migration. Clin Infect Dis.

[5] Kärki T, Napoli C, Riccardo F, Fabiani M, Dente MG, Carballo M (2014). Screening for infectious diseases among newly arrived migrants in EU/EEA Countries—varying practices but consensus on the utility of screening. Int J Environ Res Public Health.

[6] Stanaway JD, Flaxman AD, Naghavi M, Fitzmaurice C, Vos T, Abubakar I (2016). The global burden of viral hepatitis from 1990 to 2013: findings from the global burden of disease study 2013. Lancet.

[7] [Egypt] MoHaP. Egypt Health Issues Survey 2015. Cairo, Egypt and Rockville, Maryland, USA: Ministry of Health and Population and ICF International: El-Zanaty and Associates [Egypt] & and ICF International; 2015

[8] Hassanin A, Kamel S, Waked I, Fort M (2021). Egypt’s ambitious strategy to eliminate hepatitis c virus: a case study. Global Health: Sci Pract.

[9] Tsang S, Royse CF, Terkawi AS (2017). Guidelines for developing, translating, and validating a questionnaire in perioperative and pain medicine. Saudi J Anaesth.

[10] Jain S, Dubey S, Jain S (2016). Designing and validation of questionnaire. Int Dent Med J Adv Res.

[11] Obesity: preventing and managing the global epidemic. WHO obesity technical report series 894 [Internet]. World Health Organization. 2000 [cited 18 March 2022]. Available from: https://apps.who.int/iris/handle/10665/42330 11234459

[12] den Hartog G, van Binnendijk R, Buisman A-M, Berbers GA, van der Klis FR (2020). Immune surveillance for vaccine-preventable diseases. Expert Rev Vaccines.

[13] Chernet A, Utzinger J, Sydow V, Probst-Hensch N, Paris D, Labhardt N (2018). Prevalence rates of six selected infectious diseases among African migrants and refugees: a systematic review and meta-analysis. Eur J Clin Microbiol Infect Dis.

[14] Safiabadi M, Rezaee-Zavareh MS, Alavian SM (2017). Estimation of hepatitis a virus infection prevalence among eastern Mediterranean and Middle Eastern countries: a systematic review and pooled analysis. Hepat Mon.

[15] Freidl GS, Tostmann A, Curvers M, Ruijs WL, Smits G, Schepp R (2018). Immunity against measles, mumps, rubella, varicella, diphtheria, tetanus, polio, hepatitis A and hepatitis B among adult asylum seekers in the Netherlands, 2016. Vaccine.

[16] Elbahrawy A, Ibrahim MK, Eliwa A, Alboraie M, Madian A, Aly HH (2021). Current situation of viral hepatitis in Egypt. Microbiol Immunol.

[17] Kaddoura M, Allaham R, Abubakar A, Ezzeddine A, Barakat A, Mala P (2020). Hepatitis A virus genotype IB outbreak among internally displaced persons, Syria. Emerg Infect Dis.

[18] Cuthbert JA (2001). Hepatitis A: old and new. Clin Microbiol Rev.

[19] Trépo C, Chan HL, Lok A (2014). Hepatitis B virus infection. The Lancet.

[20] Hepatitis B [Internet]. WHO. 2021 [cited 28 March 2022]. Available from: https://www.who.int/news-room/fact-sheets/detail/hepatitis-b

[21] UNICEF. WHO and UNICEF estimates of national immunization coverage 6 July 2021 [Available from: https://data.unicef.org/resources/immunization-country-profiles/

[22] Poovorawan Y, Chongsrisawat V, Theamboonlers A, Crasta PD, Messier M, Hardt K (2013). Long-term anti-HBs antibody persistence following infant vaccination against hepatitis B and evaluation of anamnestic response: a 20-year follow-up study in Thailand. Hum Vaccin Immunother.

[23] Posuwan N, Vorayingyong A, Jaroonvanichkul V, Wasitthankasem R, Wanlapakorn N, Vongpunsawad S (2018). Implementation of hepatitis B vaccine in high-risk young adults with waning immunity. PLoS ONE.

[24] Pondé R (2011). The underlying mechanisms for the “simultaneous HBsAg and anti-HBs serological profile”. Eur J Clin Microbiol Infect Dis.

[25] Schweitzer A, Horn J, Mikolajczyk RT, Krause G, Ott JJ (2015). Estimations of worldwide prevalence of chronic hepatitis B virus infection: a systematic review of data published between 1965 and 2013. Lancet.

[26] Liu F, Guo Z, Dong C (2017). Influences of obesity on the immunogenicity of Hepatitis B vaccine. Hum Vaccin Immunother.

[27] El-Ghitany EM, Farghaly AG (2019). Geospatial epidemiology of hepatitis C infection in Egypt 2017 by governorate. Heliyon.

[28] UNHCR, WHO Commend Egypt’s Inclusion of Refugees in “100 Million Seha” [Internet]. 2019 [cited 9 April 2019]. Available from: https://www.unhcr.org/eg/12727-unhcr-who-commend-egypts-inclusion-of-refugees-in-100-million-seha.html

[29] Talaat M, Afifi S, Reaves EJ, Elsood HA, El-Gohary A, Refaey S (2019). Evidence of sustained reductions in the relative risk of acute hepatitis B and C virus infections, and the increasing burden of hepatitis A virus infection in Egypt: comparison of sentinel acute viral hepatitis surveillance results, 2001–17. BMC Infect Dis.

